# Green and Sustainable Ultrasound-Assisted Anodic Electrochemical Preparation of Graphene Oxide Dispersions and Their Antioxidant Properties

**DOI:** 10.3390/molecules28073238

**Published:** 2023-04-05

**Authors:** Daria-Maria V. Ratova, Ivan V. Mikheev, Grigoryi R. Chermashentsev, Konstantin I. Maslakov, Sergei Yu. Kottsov, Dmitrii N. Stolbov, Sergey V. Maksimov, Madina M. Sozarukova, Elena V. Proskurnina, Mikhail A. Proskurnin

**Affiliations:** 1 Department of Chemistry, Lomonosov Moscow State University, 1-3 Leninskie Gory, 119991 Moscow, Russia; darmarrat@gmail.com (D.-M.V.R.); chermashentsev96@mail.ru (G.R.C.); nonvitas@gmail.com (K.I.M.); stolbovdn@my.msu.ru (D.N.S.); irber@yandex.ru (S.V.M.); s_madinam@bk.ru (M.M.S.); 2 Kurnakov Institute of General and Inorganic Chemistry, Russian Academy of Sciences, 117901 Moscow, Russia; sergey12-17@yandex.ru; 3 Laboratory of Molecular Biology, Research Centre for Medical Genetics, 1 Moskvorechye St., 115522 Moscow, Russia; proskurnina@gmail.com

**Keywords:** graphene oxide, anode, graphite exfoliation, chemiluminescence, SOD-like activity, lipid peroxidation

## Abstract

A fast method for preparing aqueous graphene oxide (GO) dispersions by electrochemical oxidation of a graphite anode without preliminary intercalation with oxidizing agents is proposed. Ultrasonic probing was used in the modulation mode of ultrasonic waves (work/rest) for more efficient graphite oxidation–exfoliation. It is shown that the 4/2 s mode of ultrasonic modulation is the most effective due to the probe material’s low corrosion while maintaining the optimum synthesis temperature not exceeding 30–35 °C and achieving the best characteristics of the resulting product. Three cases of anodic oxidation of graphite to obtain graphene oxide were considered: (1) a combined cathode–anode compartment, (2) a split cathode–anode salt-bridged compartment, and (3) separated anode compartment with a 3.5 kDa dialysis membrane. It was determined that the approach to synthesis with a divided cathode–anode compartment makes it possible to obtain GO sheets with fewer defects compared to chemical methods or methods with a combined cathode–anode compartment and makes it possible to control the oxidation degree of the material (C:O ratio) by varying the current density. The prepared samples showed good stability for more than six months. The spectral and morphological characteristics were studied. Using chemiluminometry in the luminol/Co(II)/H_2_O_2_ system, the antioxidant properties concerning three key reactive oxygen species (H_2_O_2_, superoxide anion radical, and hydroxyl radical) were demonstrated. It was also shown that the prepared GO dispersions do not induce lipid and phospholipid peroxidation.

## 1. Introduction

Graphene is a two-dimensional carbon material used in various industries [1]. However, its use in medicine is severely limited by its toxicity [2] and low solubility in polar solvents. Its use as a catalyst is problematic due to the zero band gap [3]. Shifting from graphene to graphene oxide (GO) makes it possible to eliminate these problems by preserving the unique properties of the graphene conjugate network of bonds.

Graphene oxide is a two-dimensional graphene-based carbon material with sp^2^ clusters surrounded by sp^3^ carbon atoms connected to oxygen-containing groups [4]. Due to its unique properties, this material is of increasing interest in the scientific community, as it is successfully used in electrochemical sensors for hydrogen peroxide [4], glucose [5], nucleic acids [6], cholesterol [7], and other biologically significant molecules, as well as in optical biosensors [8]. GO makes it possible to detect and visualize cancer cells, e.g., using GO nanosheets in vivo [9] and nanoribbons, which are used in photothermal therapy [10]. Currently, the enzyme-like nanozyme properties of GO [8] and its uses as a regulator (scavenger) of biologically significant free radicals are actively being investigated [11]. Chemically prepared carboxyl-modified graphene oxide (GO–COOH) possesses intrinsic peroxidase-like activity [12]; the green method for the reduction of graphene oxide by a seed extract from *Punica granatum* L. (pomegranate) shows biocompatibility and excellent radical-scavenging activity against 2,2-diphenyl-1-picrylhydrazyl free radicals [13].

GO synthesis approaches have been developed, mainly for the chemical oxidation of graphite. These methods were proposed by Brodie [14] and Hummers [15], as well as their improved modifications [16,17]. The main disadvantages of chemical oxidation methods are associated with the release of toxic gases, such as NO_x_ or ClO_x_; thus, a topical task is to find safer and greener approaches to produce GOs and their aqueous dispersions.

A promising green and sustainable way to prepare GO is the electrochemical preparation–exfoliation of graphite flakes with additional exfoliation by ultrasound. Electrochemical exfoliation of graphite can be carried out by anodic and cathodic methods [18,19] depending on the type of intercalation ions to prepare electrochemically generated graphene oxide (EGO). In these processes, the critical factor is the intercalation of an electrolyte into the graphite layer, which leads to the exfoliation of the graphite structure with subsequent stratification into separate sheets [20]. The most suitable and studied electrolyte is (NH_4_)_2_SO_4_ with the same cation and anion mobility [21], which promotes depolarization and exfoliation of the graphite surface due to the nucleophilic reaction at the edges and boundaries of graphite grains. This expansion facilitates the easy embedding of ${SO}_{4}^{2-}$ ions between the expanded graphite layer, facilitating delamination [22,23].

Ultrasonic (US) treatment finds its application in green chemistry [24]. Ultrasonication for the exfoliation and solubilization of various materials, including graphene [25], fullerenes [26], and carbon quantum dots [27], has been used widely. Ultrasonic treatment in the electrochemical synthesis of GO contributes to a more efficient detachment of graphite flakes in the ultrasonic field [28] and the effective formation of GO dispersions, especially aqueous dispersions [29]. Ultrasonic cavitation microbubbles produce high-speed jets of microfluid and shock waves, causing shear forces that fragment graphite flakes and delaminate individual graphene sheets [30]. Two US sources are most common: (1) baths and (2) high-energy US probes [31]. The choice of an ultrasonic device is essential for the most effective layering of the material; in this regard, US probes are more effective since they allow for the power control of ultrasonic waves, frequency, and processing time [30,32]. The main disadvantages of US probes are uncontrolled heating of the system and significant morphological changes in the GO structure. Heating reduces the current and current density and, as a result, lowers the synthesis efficiency [33] and decreases the total yield by raising the number of defects, which can include edge defects or oxygen functional groups, from 25 to 95 °C [34]. Furthermore, during ultrasonic treatment, the probe material is destroyed and corrodes with the formation of titanium (di)oxide particles [35], which complicates the following purification and use of EGO for biomedical purposes [36].

In recent decades, the development of nanopharmaceuticals that can regulate redox homeostasis in cells and tissues has reached the threshold of practical implementation. The ability of nanomaterials to initiate cellular signals leading to oxidative stress has become the leading hypothesis in nanotoxicology [37]. However, an alternative property of nanomaterials also manifests itself: they potentially reduce oxidative stress quantitatively by decreasing the level of reactive oxygen species (ROS) [38], which include superoxide anion radicals (SARs), hydrogen peroxide, hydroxyl radicals, and singlet oxygen [39]. However, there is still a need to understand the mechanisms that determine the biocompatibility or toxicity of nanomaterials to human cells and tissues. Aqueous dispersions of high-purity graphene oxide are of considerable interest to assess the potential effect on morphological and structural changes in the cell membrane and their activity in the body. The absence of contaminants (components of oxidants used in the synthesis), which may be involved in the metabolic process, can reduce the cytotoxicity of potential nanopharmaceuticals based on them.

This work aims to develop a method for ultrasonic anodic electrochemical oxidation–exfoliation of graphite to obtain stable graphene oxide aqueous dispersions and test their antioxidant capacity in model systems by chemiluminometry.

## 2. Results and Discussion

Briefly, the results can be summarized as follows.

1. The conditions for the preparation and separation of aqueous dispersions of electrochemically generated graphene oxide (EGO) via anodic oxidation of graphite under the application of additional ultrasonic exfoliation of graphite under pulsed (modulation) conditions were determined.
2. The conditions for isolation and purification of the prepared EGO with a dialysis bag (membrane) from (a) the electrolyte used in the synthesis and (b) formatted byproducts during electrolysis were determined.
3. For the developed graphite anodic oxidation synthesis methods, many colloidal stability and morphology parameters are accurately reproducible. Additional structural and morphological information about the synthesized samples was obtained from the data of (a) molecular spectroscopy methods, as well as FTIR and Raman spectroscopies; (b) X-ray photoelectron spectroscopy; and (c) SEM and TEM. Dynamic light scattering (DLS) was also used to determine lateral particle sizes and zeta potentials.
4. A chemiluminometry assay was used for electrochemically synthesized graphene oxide samples to evaluate their reactivity concerning reactive oxygen species (H_2_O_2_, superoxide anion radicals, and hydroxyl radicals) and to assess the induction of lipid/phospholipid peroxidation.

### 2.1. General Concept of EGO Synthesis 

Six samples of EGO were prepared (procedures 2–6, see Materials and Methods section, Section 3.8), three of which are the most representative with the best stability, concentration properties, and characteristics, namely samples EGO 2 (procedure 3), with a high amount of >C=C< bonds and oxide groups; EGO 5 (procedure 5), with a high quantity of >C=C< bonds and the presence of oxide groups; and EGO 6, which was obtained under more harsh electrochemical conditions (method 6), for comparison sake.

**Figure 1:**
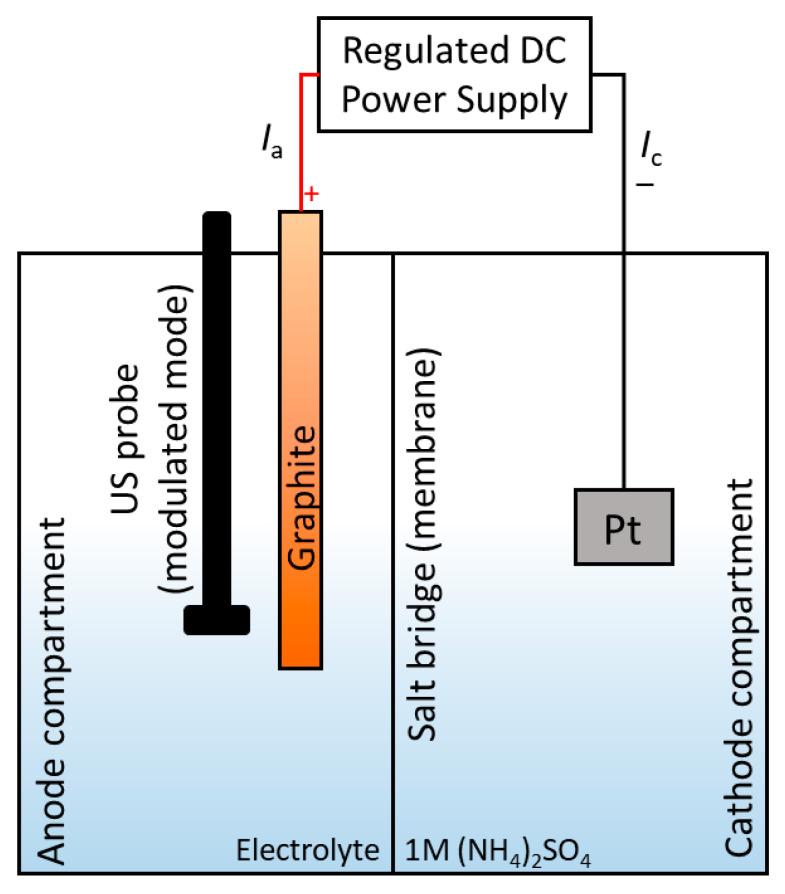
Typical scheme of the experimental setup for obtaining electrochemically generated graphene oxide.

Three types of setups were considered for anodic oxidation of graphite to produce graphene oxide (Figure 1): (1) combined cathode–anode compartment; (2) separated cathode–anode compartment with a salt bridge; and (3) separated anode compartment with a 3.5 kDa dialysis membrane (bag). Generalized synthesis conditions are given in Table 1. Specific attention should be paid to the choice of the main electrochemical parameters of the electrolysis process: the current and the associated current density value, voltage, and electrolysis time (Table 1).

At the same time, cathodic and anodic electrochemical processes in the presence of ammonium sulfate (NH_4_)_2_SO_4_ can be expressed by the following equations describing the electrolysis of water in an electrochemical cell and electrolyte reactions:

I. At the cathode:

- 2H_2_O + 2$\overset{-}{e}$ = H_2_ + 2OH^−^ (–0.828 V) (1); the formation stage of hydrogen, which can potentially reduce EGO to a reduced sample of (rEGO) only in the case of a combined cathode–anode compartment.

II. At the cathode:

- 2H_2_O – 4$\overset{-}{e}$ = O_2_ + 4H^+^ (1.229 V) (2); water oxidation;
- >C=C< → >C=O, –COOH, >CH–OH, >C=O etc. (3); graphite structure oxidation [40];
- 2${SO}_{4}^{2-}$– 2$\overset{-}{e}$ = $S_{2}O_{8}^{2-}$ ($E_{S_{2}O_{8}^{2-}/{SO}_{4}^{2-}}$=2.010 V) (4); the formation of an additional oxidizer peroxydisulfate ion $S_{2}O_{8}^{2-}$ in solution.

III. Reactions of hydrolysis of byproducts:

- (NH_4_)_2_S_2_O_8_ + 2H_2_O = 2(NH_4_)HSO_4_ + H_2_O_2_ (5); hydrolysis of electrolysis byproducts.

In [41], the high oxidizing ability of the sulfate radical (SO_4_^–^∙) anodically formed from SO_4_^2−^, was shown. In addition, peroxydisulfate ions, because of the recombination of the sulfate radical (SO_4_^−^∙), can increase the oxidation efficiency of the graphite anode due to the formation of other sulfate radicals.

In all cases, the anode was immersed in 10 cm of the electrolyte solution; in an additional stage, when the platinum wire was the anode, it was immersed in 15 cm of solution. This allowed us to estimate and compare the current density under different synthesis conditions (Table 1). It is worth noting that the value of the current density may correlate with the porosity of the anode material, as well as its mass. In some cases, when the anode was stratified quickly enough (procedures 2 and 3), we applied additional current to the electrochemical cell at a constant potential. In this case, the platinum wire was selected as the anode, and the current density increased six times (Table 1). Previously, this approach was not used for the electrochemical synthesis of aqueous GO dispersions. Replacing the electrochemical regime in the cell (voltage and current density) contributed to increased product yield. Furthermore, according to the experimental data of procedures 4 and 5, introducing a salt bridge causes an additional drop in current density due to the appearance of extra electrical resistance in the system. 

Finally, the electrochemical oxidation of the EGO preparation stage was carried out, and dialysis purification (procedure 8) was conducted using a 3.5 kDa dialysis membrane. The mother liquor was purified for removal of (1) the electrolyte used in synthesis, (2) metal ions obtained from US probe corrosion, and (3) byproducts of graphite anode oxidation (oxidative debris) [36], which were additionally formed as a result of ultrasound treatment [42].

### 2.2. Selection of the Ultrasonic Treatment Mode

The ultrasonic treatment mode plays a crucial role in the effective separation and dispersion of graphene oxide in water [43]. Due to system overheating with continuous exposure, the reproducibility of ultrasonic treatment decreases, and the liquid evaporates uncontrollably [44]; therefore, many studies recommend thermostatic conditions or ice additives [25]. This work considers two operation modes of the ultrasonic probe: (1) continuous and (2) pulsed modulation. The ultrasonic setup was modified with an additional module that allowed the experiment to be carried out in a modulation mode, which made it possible to generate variable ultrasonic radiation in the range of 0.1–9.9 s (*t*_work_) with the specified rest time parameters (*t*_rest_), i.e., 10 s (*t*_work_ + *t*_rest_). 

An experiment assessed the degree of erosion of the titanium ultrasonic probe material depending on the time (number of modulations, procedure 1). In all experiments, the total US treatment time of deionized water was 30 min. At the same time, the total number of modulations varied from 200 to 900, corresponding to modulation modes of working/rest times from 10/1 to 1/5 of the US probe (Table 2).

**Figure 2:**
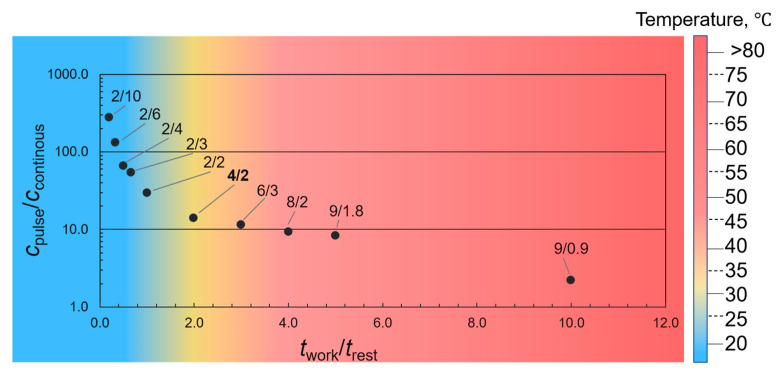
The concentration of Ti in the modulation mode normalized to the continuous mode. The temperature measurement error is about ±5 °C.

The degree of corrosion was assessed by ICP–AES to select the best practice for conducting EGO synthesis with the minimum erosion of the US probe. The dependence of the amount of Ti (ppm) on the modulation mode is depicted in Figure 2; here, analytical signals are normalized values (the behavior of Al is similar):

(1) $$Analytical\;signal={c(Ti)}_{pulse}/{c(Ti)}_{continuous}$$

Analytical signal is the titanium amount in the continuous operating US mode normalized to titanium amount in the pulsed US mode. As a result, the constant mode provides the minor destruction of the probe and the minimum degree of titanium contamination of the sample. Still, it significantly warms up the system, reducing the current density during synthesis.

Analysis of the data in Figure 2 shows that the modulation (pulsed) mode provides a significant overheating of the system (>35 °C) with a modulation mode of 6/3 s or more. At the same time, the degree of erosion of the probe material reaches a plateau. As a result, a modulation mode of 4/2 s was selected to achieve the lowest degree of corrosion (*c*_Ti_ = 14.3 ppm, *c*_Al_ = 21.1 ppm), while the temperature did not exceed 35 ± 5 °C. These data correlate with the previously known values but did not coincide precisely [21], so an ultrasonic treatment mode with work/rest of 2/4 s was selected. It is suggested that the optimal synthesis temperature is maintained under this mode. In addition, pulse processing modes such as 2/2, 2/4, and 2/8 s were tested. However, the probe destruction degree in pulsed mode for EGO synthesis has not been studied previously.

### 2.3. Characterization of Prepared EGO

#### 2.3.1. Spectroscopic and Morphological Properties

- *ATR-FTIR spectroscopy*

ATR-FTIR spectra were recorded to estimate the variation of the oxygen groups in the graphene oxide structure. Below are the results obtained for three samples under different conditions of anodic oxidation, which differ significantly from each other.

All the main FTIR bands coincide with those previously known for graphene oxide. To obtain the finest FTIR spectra from aqueous EGO dispersions, we placed samples into ATR diamond crystal at 50 °C to evaporate water, which led to the formation of a thin film of the sample and better adhesion to the crystal surface in comparison with standard application and pellet pressing, as previously discussed in [45]. 

The broad absorption band in the 3600–2400 cm^−1^ region includes the absorption bands of water [46], fragments of *ν*_O–H_ (3300 cm^−1^), and *ν*_C–H_ (–CH at 2925 cm^−1^ and –CH_2_ at 2847 cm^−1^). All samples have absorption bands of interplane H_2_O (1633 cm^−1^), which are often incorrectly interpreted as C=C absorption bands [46]; however, reliable experiments with isotopic D_2_O exchange show that this band corresponds to H_2_O molecules embedded in graphite layers [47]. Overlapping bands of oxygen groups are also observed in the fingerprint region, which belongs to *ν*_C=O_ (1737 and 1113 cm^−1^), *β*_O–H_ (C–OH at 1388 cm^−1^), *ν*_C–O–C_ (1200 cm^−1^), and *ν*_C–O_ (1048 cm^−1^). There are also absorption bands of aromatic *ν*_C=C_ (1580 cm^−1^), *β*_C–H_ (1450 cm^−1^), and *ν*_C–C_ (900 cm^−1^) [47].

**Figure 3:**
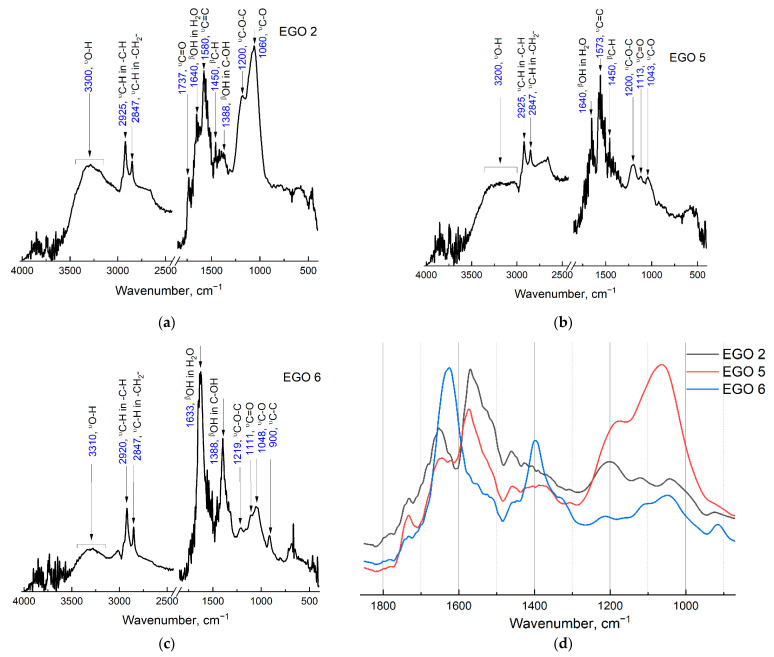
ATR-FTIR spectra of samples: (**a**) EGO 2, two-step synthesis using a separated cathode–anode compartment and additional current transmission using platinum rods as cathode and anode (procedure 3); (**b**) EGO 5, one-step synthesis with a separated cathode–anode compartment without additional current treatment using a platinum rod as an anode (procedure 5); and (**c**) EGO 6, combined cathode–anode compartment (procedure 6). Spectra a, b, and c were recorded in the absorption mode from 4000 to 400 cm^−1^. (**d**) Combined normalized figures for all three EGO samples.

During the experiment, to obtain a sample of EGO 2, it was possible to increase the number of oxygen groups by additional current treatment of EGO already exfoliated in the separated cathode–anode compartment. The absorption bands of *ν*_C–O–C_ and *ν*_C–O_ significantly increased, and the >C=O absorption band also appeared at 1737 cm^−1^, while the intensity of the aromatic >C=C< band remained. Thus, the preparation method corresponding to procedure 3 provides GO without extra significant defects (Figure 3a).

In the separated cathode–anode compartment (EGO 5), the current is significantly less than in the combined compartment; however, there are also many absorption bands of oxygen groups, as well as an intense absorption band of aromatic >C=C<, which indicates a milder oxidation with the preservation of a significant part of the graphene-like structure (Figure 3b).

Based on the spectra (Figure 3c), it can be seen that for considerable currents (over 2.5 A), the sample (EGO 6) has a significantly larger number of oxygen groups; however, the intensities of the bands have minor values, and there are no aromatic >C=C< bands, which may indicate excessively rapid destruction of the electrode, which does not allow for additional oxidation of the resulting GO and destruction of the graphene-like structure due to the parallel process of cathodic reduction in the combined cathode and anode space [48], especially hydroxyl and epoxy groups [49] (Figure 3d).

- *UV/vis spectroscopy*

**Figure 4:**
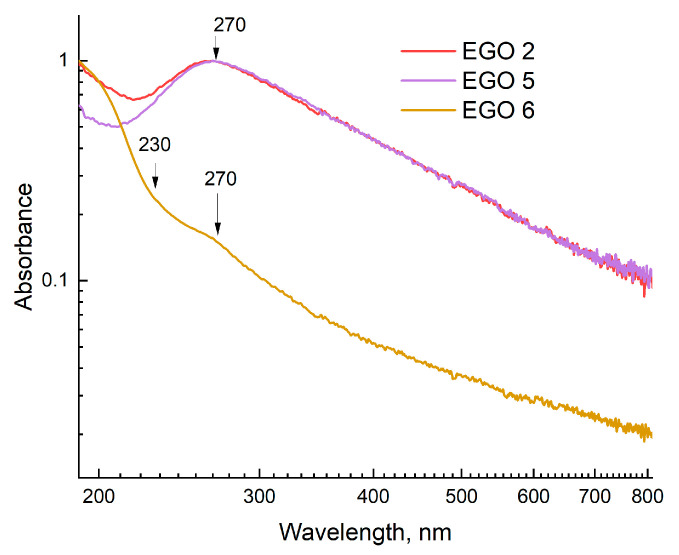
Absorption spectra of samples: EGO 2, two-step synthesized samples of EGO with a separated cathode–anode compartment with additional current treatment and a platinum rod as an anode (procedure 3); EGO 5, one-step synthesized samples of EGO with a separated cathode–anode compartment without further current treatment (procedure 5); and EGO 6, one-step synthesized samples of EGO with a combined cathode–anode compartment (procedure 6); path length is 1 mm.

UV-visible absorption spectra in the range of 190 to 800 nm were recorded (Figure 4). Graphene oxide has two distinctive features: (1) a primary absorption peak at 230 nm, which can be attributed to the π–π*-transitions of >C=C< in the system of aromatically bonded carbon rings; and (2) a wide shoulder with a maximum at ~300 nm, which is usually associated with n–π*-transitions of >C=O [50].

However, this absorption band can also be attributed to optical transitions between π and π* states in molecular sp^2^ domains of finite size and sp^2^ clusters of several nanometers in size [51]. When reducing graphene oxide, the peak at 230 nm is red-shifted towards ca. 270 nm, the shoulder at 300 nm disappears, and the total absorption in the near-IR range increases significantly due to the removal of functional groups from the primary graphite plane and the restoration of its conjugate structure [52].

Thus, based on the obtained absorption spectra and existing data, it can be concluded that the EGO 6 sample corresponds mostly to GO, while EGO 2 and EGO 5 samples are more like reduced GO (rGO). In addition, it was shown in [53] that ultrasound probes can cause more severe damage to the structure and lead to the loss of a more significant number of functional groups. Therefore, UV/vis spectroscopy can be positioned to rapidly monitor the quality and screening of the resulting GO products.

- *Raman spectroscopy*

**Figure 5:**
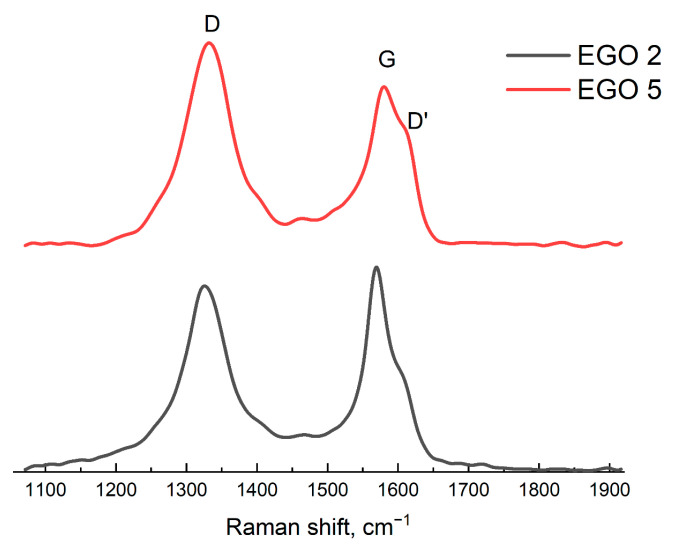
Raman spectra for samples: EGO 2 (black line), two-step synthesized samples of EGO with a separated cathode–anode compartment with additional current treatment and a platinum rod as an anode (procedure 3); EGO 5 (red line), one-step synthesized samples of EGO with a separated cathode–anode compartment without further current treatment (procedure 5).

Figure 5 shows the Raman spectra of the EGO 2 and EGO 5 samples. The spectra show D and G bands with centers at about 1325 and 1580 cm^−1^, respectively [54]. Raman spectroscopy data confirm the results of ATR-FTIR spectroscopy (Figure 3) and the assumption that EGO 2 is natural graphene oxide and EGO 5 is reduced graphene oxide (rGO). Both G and D vibrational bands of sp^2^ carbon are present [55]. The ratio of bands (*I*_D_/*I*_G_) corresponds to the behavior of GO and rGO [56].

- *Morphology of prepared samples by scanning electron microscopy*

**Figure 6:**
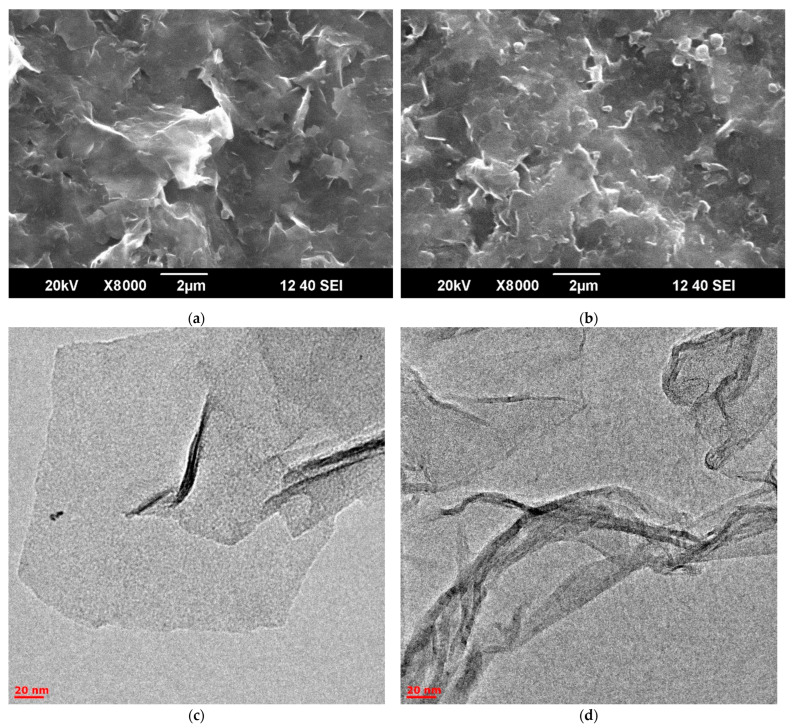
SEM images: (**a**) EGO 2, two-step synthesized samples of EGO with a separated cathode–anode compartment with additional current treatment and a platinum rod as an anode (procedure 3); (**b**) EGO 5, one-step synthesized samples of EGO with a separated cathode–anode compartment without further current treatment (procedure 5). HRTEM images: (**c**) EGO 2; (**d**) EGO 5.

The graphene oxide sheet size and the prepared EGO morphology were determined using SEM (Figure 6a,b) and HRTEM (Figure 6c,d). According to these images, the morphology is uncharacteristic of graphene oxide [57]. This may be due to particle coagulation during evaporation from the solution to prepare the sample for measurements [58].

- *X-ray photoelectron spectroscopy for C:O ratio estimation*

**Figure 7:**
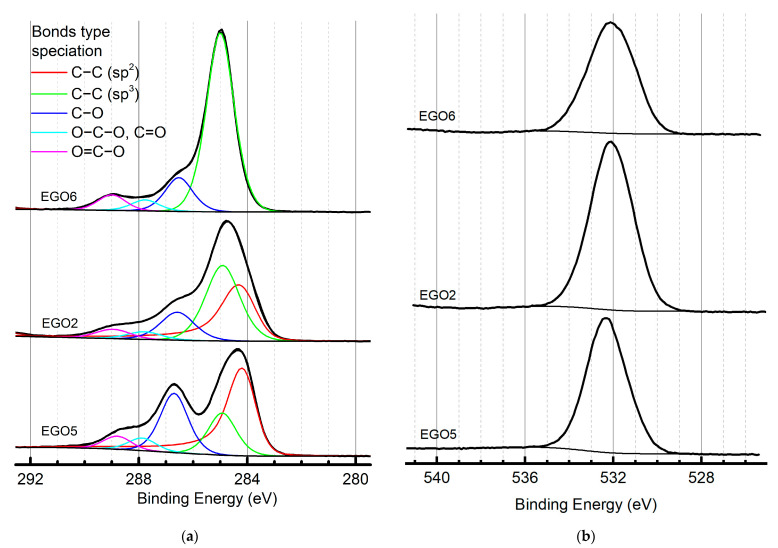
XPS spectra of the prepared EGO samples: (**a**) C 1s; (**b**) O 1s.

Deconvolution and interpretation of the results were carried out as published elsewhere [59]. C 1s XPS spectra (Figure 7) were deconvoluted into components corresponding to different states of atoms. O 1s spectra are observed as broad bands with weakly resolved states, which makes it challenging to reliably deconvolute them into components.

According to existing data, the C:O ratio for standard preparation methods of graphene oxide may vary in a wide range due to several successive oxidation stages: Hummers’ and Brodie, 2.4 ÷ 3.3 [60]; Staudenmaier, 2.2 ÷ 3.0 [16]; and Hofmann, 2.5 ÷ 3.0 [61]. For reduced graphite oxide (rGO), a C:O ratio of more than 5, 6.8 ÷ 8.3 [62] or 5.2 ÷ 6.4 [63] was reported. In this work, a ratio of three to four was estimated for the prepared EGO samples, which is close to that of conventional chemical oxidation preparation procedures (Table 3). XPS data (Figure 7) correlate with ATR-FTIR spectra (Figure 3). In Figure 3, EGO 2 has the best ratio of C=C bonds and oxide groups, while in EGO 5, this ratio is much higher. The XPS spectra for EGO 6 do not show C=C bonds, which also correlates with the ATR IR data.

#### 2.3.2. Quality of Prepared EGO Products (Stability and Concentration Parameters)

The samples have a bimodal particle size distribution consistent with the polydispersity index value. Usually, the lateral dimensions for graphene oxide obtained by chemical means range from 0.7 to 1 µm [64], but in this study, we obtained precise, reproducible results with a fine fraction (on average, up to 300 nm). The main results and characteristics of the obtained products are shown in Table 4. The product yield was estimated based on the total organic carbon data. The average zeta potential shows that such graphene oxide nanoparticles have good stability [65]. According to zeta potential values, EGO 2 exhibited agglomeration threshold behavior (agglomerates of 2–10 particles), and EGO 5 and 6 maintained a plateau of slight stability (few agglomerates). The concentrations are suitable for further biomedical application and testing [66].

The total yield of graphene oxide has an average value of ca. 10%, which is lower than that reported in [34]. In [34], deionized water and 50 V had applied bias for one hour at room temperature for EGO preparation. In contrast, we proposed a much more cost-effective approach using lower voltage parameters (<30 V).

### 2.4. Chemiluminescence Assay

Having obtained aqueous dispersions of graphene oxide (EGO) and carried out their complete characterization by spectroscopy and microscopy, we studied their behavior in vitro in a chemiluminometry assay (CL) in the following models. 

(1) The enzyme-like activity of EGO samples in the system of Co(II), H_2_O_2_, and luminol: Besides H_2_O_2_, this system is a source of SAR and hydroxyl radicals [67] (procedure 9). Previously, we developed a method using this system to assess the activity of superoxide dismutase (SOD) and SOD mimics [68]. Here, we use luminol as an enhancer sensitive to all formed free radical species, including SAR [69,70], to assess total ROS.

(2) A lipid peroxidation model that is phosphatidylcholine or linoleic acid hydroperoxide in the presence of coumarin-334 (procedure 10): The mechanism of lipid peroxidation has long been well-studied [71]. For its quantitative assessment, the determination of lipid hydroperoxides using chemiluminescence with coumarins is used [72]. Phospholipid peroxidation is less studied but is believed to be based on a similar mechanism [73,74,75]. Coumarin-enhanced chemiluminescence can also be used to research phospholipid peroxidation [76]. Here, we use original specially designed chemiluminescent protocols to determine lipid and phospholipid hydroperoxides.

#### 2.4.1. Reactivity towards ROS

**Figure 8:**
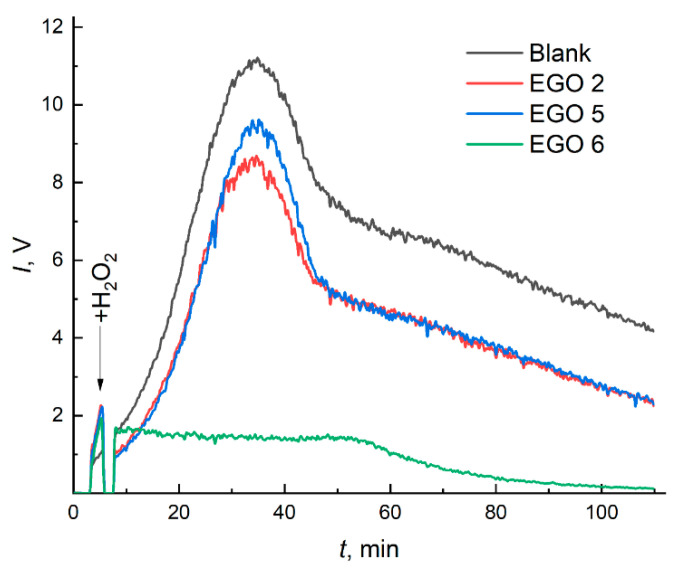
Chemiluminograms of EGO aqueous dispersions in the CL system: EGO 2, two-step synthesized samples of EGO with a separated cathode–anode compartment with additional current treatment and a platinum rod as an anode (procedure 3, *c*_EGO_ = 16.0 ± 2.0 ppm); EGO 5, one-step synthesized samples of EGO with a separated cathode–anode compartment without further current treatment (procedure 5, *c*_EGO_ = 15.2 ± 1.8 ppm); and EGO 6, one-step synthesized samples of EGO with a combined cathode–anode compartment (procedure 6, *c*_EGO_ = 16.8 ± 2.5 ppm). The CL system contained luminol (100 µL, 1 mM), Co(II) (65 µL, 1 mM), and H_2_O_2_ (40 µL, 1100 mM). The tested systems were spiked with 200 µL of each EGO sample.

Chemiluminograms recorded for mixtures containing aqueous EGO dispersions and Co(II)/H_2_O_2_/luminol are shown in Figure 8.

Analysis of Figure 8 shows that EGO exhibits antioxidant properties towards ROS, apparently due to oxygen groups on the surface [77]. Signal suppression for EGO at a concentration of ca. 15 ppm shows EGO 6 > EGO 5 ≥ EGO 2. It should be noted that with an increase in the number of oxygen groups, antioxidant properties are enhanced. The EGO 6 sample, in turn, suppresses chemiluminescence significantly (up to 85%); this may occur due to the presumed absence of >C=C< bonds in this sample (FTIR data, Figure 3), which demonstrates their ROS scavenging ability. Additionally, the concentration of half-suppression (*c*_1/2_) of the signal of a blank experiment for the EGO samples was (ppm): EGO 6, 10; EGO 5, 35; EGO 2, 60; for SOD, the *c*_1/2_ was 130 nM.

The unsolved issue for graphene oxide research is the molar concentration of GO due to the unknown composition C_x_H_y_O_z_ and the indefinite quantity of oxygen-containing addends. Still, only the C:O ratio [78] and the concentration of carboxyl groups can be estimated by titration [79], which may play a crucial role. However, there is no detailed mechanism of reactivity of graphene oxide towards ROS, etc. The estimations of antioxidant (scavenging) ability reported in this work are still based on mass concentrations.

#### 2.4.2. Pro-Oxidant Activity EGO towards Lipids and Phospholipids

**Figure 9:**
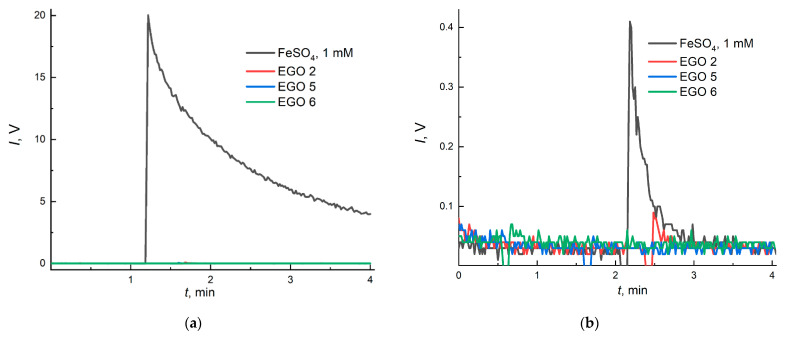
Chemiluminograms of peroxidation (**a**) lipids in a system of linoleic acid (5 mM, 25 µL), coumarin C-334 (5 mM, 10 µL), and Fe(II) (20 mM, 50 µL); (**b**) phospholipids in a system of phosphatidylcholine hydroperoxide (28 mM, 50 µL), coumarin C-334 (5 mM, 10 µL), and Fe(II) (20 mM, 50 µL). In all cases, ca. 3.5 ppm EGO was added (50 µL).

Lipids and phospholipids are key components in cells. They modulate the permeability of membranes, act as their supporting framework, and participate in signal transmission in response to external and internal stimuli [80]. Therefore, the biochemical activity of EGO concerning lipids and phospholipids was investigated. An aqueous Fe(II) salt solution was used as a lipid or phospholipid peroxidation reference inducer. Adding purified EGO aqueous dispersions to a solution of phosphatidylcholine or linoleic acid hydroperoxide and coumarin C-334 (a chemiluminescence enhancer) did not increase chemiluminescence, which indicates the absence of pro-oxidant activity of EGO with respect to lipids and phospholipids (Figure 9).

Summing up the results of chemiluminescent studies, EGO aqueous dispersions are characterized as antioxidants towards the key ROS (superoxide anion radicals, hydrogen peroxide, and hydroxyl radicals). At the same time, they do not have a pro-oxidative effect concerning lipid peroxidation and phospholipid peroxidation. These conclusions demonstrate the safety of EGO aqueous dispersions concerning these parts of ROS metabolism and expand the potential application of EGO in biomedicine.

## 3. Materials and Methods

### 3.1. Equipment for the Experimental Setup for Ultrasonic Anodic Oxidation–Exfoliation of Graphite

The setup included a metallic platinum cathode (area, 9 cm^2^), a graphite anode (*d* = 3.2 and 2 mm, Faber Castel HB type, Nürnberger, Germany), an MS-3010D power supply (DC source) (Maisheng, Zhengzhou, China; operating conditions: up to 30 V, 10 A), a copper conductor equipped with crocodile clips (operating up to 1000 V, 10 A), a magnetic stirrer, and salt bridges (if necessary). A platinum rod with a *d* = 1 mm was used for some stages of anodic oxidation (Figure 1).

To separate the cathode–anode compartments, the following equipment was used: (1) a salt bridge consisting of borosilicate glass “U” tubes with outer diameters of 0.53 in. and inner diameters of 0.41 in. equipped with Schott filters (from 1 to 1.6 µm) at the end of the tips filled with 3.5 M KCl aqueous solution (Eisco™, Victor, NY, USA) or (2) cellulose dialysis membranes with a molecular weight cutoff of 3.5 kDa (Spectra/Por Spectrum Labs, Thermo Scientific, Waltham, MA, USA).

A mass-produced ultrasonic probe with an MEF93.T timer (LLC MELFIZ-ultrasound, Moscow, Russia) equipped with an additional unit for modulation generation of ultrasound waves was used. The ultrasonic probe had an operating frequency of 22.00 ± 1.65 kHz and operated in a pulsed mode of generating ultrasonic waves and a continuous mode [81]. An ultrasonic tip with a total surface area of 6.61 cm^2^ was used, which provided an intensity range of up to 250 W/cm^2^ in the 0.6 kW electric power mode. The ultrasonic probe was made of TM3 titanium alloys according to ISO 28401:2010. 

### 3.2. Reagents

All reagents were chemically pure or of pure grade and used as received without preliminary purification. The following reagents were used for preconditioning of the dialysis membrane from transition metal impurities: (1) hydrogen peroxide (H_3_PO_4_-stabilized, pure assay by titration 60%) (Peroxide Ltd., Moscow, Russia); (2) ethylenediaminetetraacetic acid (EDTA) (ACS reagent grade 98%, Merck, Darmstadt, Germany); and (3) ultrapure Milli-Q^®^ water (18.2 MΩ·cm at 25 °C with a TOC level of less than 3 ppb) (Millipore Corp., Darmstadt, Germany).

The 100 mM phosphate buffer solutions (PBS) with pH 7.4 and 8.6 were prepared by dissolving KH_2_PO_4_ (Sigma-Aldrich, St. Louis, MO, USA) in 1.00 L of ultrapure water, followed by the adjustment to the required pH value using granulated potassium hydroxide or concentrated hydrochloric acid (Sigma-Aldrich, St. Louis, MO, USA).

CoCl_2_ × 6H_2_O of chemically pure grade (LLC Ruskhim, Moscow, Russia), Fe(II) standard reference material (1 g/L) (LLC EcoAnalytica, Moscow, Russia), superoxide dismutase from bovine erythrocytes (Sigma-Aldrich, St. Louis, MO, USA), CL probe coumarin 334 (Sigma-Aldrich, St. Louis, MO, USA), ACS reagent-grade DMSO (MP Medicals, Solon, OH, USA) and Phospholipovit were prepared by dissolving an emulsion containing 90% phosphatidylcholine and 10% maltose in a phosphate buffer solution (Institute of Biomedical Chemistry of RAS, Moscow, Russia).

### 3.3. Characterization of Aqueous Graphene Oxide Dispersions

UV/visible absorption spectra were recorded using a Cary 4000 scanning double-beam spectrophotometer (Varian, Mulgrave, Australia). Spectrophotometric measurements were carried out in “Agilent Quartz Cuvettes” with an optical path length of 1 mm and a registration pitch of 1 nm in the 190–800 nm range. The registration of ATR spectra with Fourier transform was carried out using a Vertex 70 (Bruker Optik GmbH, Ettlingen, Germany) in the middle IR region from 400 to 4000 cm^−1^ using a MIRacle single-reflection ATR attachment with a diamond crystal (Pike, Madison, WI, USA). The EGO was separated from the graphite part using an Armed 80-2S centrifuge (LLC Armed, Moscow, Russia).

### 3.4. Chemiluminescent Models for Assessing the Antioxidant Potential of Aqueous Dispersions of Graphene Oxide

Pro- and antioxidant activities were studied by enhanced chemiluminescence in luminol/Co(II)/H_2_O_2_ systems and with lipid and phospholipid hydroperoxides in the presence of coumarin-334. Chemiluminescence measurements were carried out on (a) a Lum-1200 12-channel chemiluminometer and (b) a Lum-100 single-channel chemiluminometer (DISoft, Moscow, Russia). Chemiluminometers register visible light in the range of 300–700 nm; bandpass filters were not used. Signal processing was performed using PowerGraph software v.3.3.11 Professional (DISoft, Moscow, Russia).

### 3.5. Elemental Analysis

An Agilent 720 ICP-OES spectrometer with an axial view (Agilent, Mulgrave, Australia) was used for the elemental analysis of impurity components. Atomic emission lines were selected based on the recommendations of ISO 11885:2007. A total organic carbon analyzer (TOC Elementar, Hamburg, Germany) was used for the main component. 

### 3.6. Surface Analysis and Morphology

The morphology and structure of the samples were studied using a JEOL JSM-6390 LA scanning electron microscope (SEM) and a JEM 2100 F/Cs transmission electron microscopy (TEM) (JEOL, Tokyo, Japan). For SEM, a sample was placed on a piece of conductive aluminum tape, then pumped and studied at an acceleration voltage of 20 kV. A grinded sample was deposited on a polymer-coated TEM copper grid from the aqueous dispersion for TEM analysis. An acceleration voltage of 200 kV was used for this analysis. An Axis Ultra DLD XPS (X-ray photoelectron) spectrometer (Kratos Analytical, Manchester, UK) was used to estimate the C:O ratio of the prepared EGO.

Raman measurements were performed with a SOL Instruments Confotec NR-500 spectrometer (Confotec, Minsk, Belarus) using a laser with a wavelength of 633 nm (16 mW power on the sample), a diffraction grating with a resolution of 1200 lines per mm, a 40× objective lens (focal length, 0.4 mm; NA, 0.75), and accumulation time of 20 s, with averaging over three measurements. ATR-FTIR spectra for EGO were recorded on a Bruker Vertex 70 single-beam IR Fourier spectrometer (Bruker Optik GmbH, Ettlingen, Germany) equipped with a GladiATR™ (PIKE Technologies, Fitchburg, WI, USA) monolithic diamond ATR for the entire spectral range from 4000 to 400 cm^−1^.

### 3.7. Processing of Data and Analysis Results

All statistical analyses were performed and all graphic illustrations were generated using Origin 2017 SR1 b9.2.257 (OriginLab Corporation, Northampton, MA, USA).

### 3.8. Procedures

#### 3.8.1. Procedures for Optimum Ultrasonic Treatment Condition Findings

Procedure 1. Selecting the mode of ultrasonic modulation of the ultrasonic probe by ICP–AES.

An ultrasonic probe was immersed in a polymeric Erlenmeyer flask filled with 30 mL of deionized water. The mode of ultrasonic radiation was chosen in the range of 0.1–9.9 s. Sonication was carried out in 200 to 900 modulations per experiment. Continuous operation was implemented with a total sounding time of 30 min for each experiment. Next, an aliquot (10 mL) of the prepared solutions was taken and acidified by concentrated nitric acid (0.15 mL), and ICP–AES was used to analyze the elemental composition.

For procedures 2–6, the final stages were always applied. EGO dispersions were separated from soggy graphite particles by centrifugation in 15 mL polymer tubes (30 min, 4000 rpm). Purification was carried out by dialysis for three days (procedure 8). 

**Attention!** For procedures 2–6, since the ultrasonic probe is immersed in a conducting solution, all the main elements of the setup, the ultrasonic probe, and the DC voltage source must be grounded to prevent short circuits.

#### 3.8.2. Procedures Describing EGO Preparation Peculiarities 

Procedure 2: Preparation of aqueous graphene oxide dispersions by anodic oxidation in the combined cathode–anode compartment using pulsed ultrasonic modulations. 

The synthesis was carried out in two steps. The anode for step 1 was graphite, and for step 2, the anode was a platinum rod. Before the start of the experiment, graphite layers were exfoliated by activation with an ultrasonic probe in a continuous mode for 10 min. Step 1: Current (2.17 A at 10.2 V) was applied through the graphite anode for 15 min until the electrode was wholly soaked to obtain graphite oxide flakes. Step 2: Next, the anode was replaced with a platinum rod, and the resulting dispersion was exposed to a current (3.37 A at 8.25 V) for 75 min to obtain fine flakes with a higher degree of oxidation; ultrasonic treatment was used throughout all stages in the work mode (4 s) and after the rest mode (2 s) and maintained the temperature within the range of 25–30 °C. The total volume of the electrolyte used in the synthesis was 800 mL.

Procedure 3: Preparation of aqueous graphene oxide dispersions by anodic oxidation in separate cathode and anode compartments with pulsed ultrasonic modulations. 

The synthesis was carried out in two steps. The anode for step 1 was graphite, and for step 2, the anode was a platinum rod. Step 1: The current (0.53 A at 15.4 V) was supplied through a graphite electrode beforehand and placed into a dialysis bag (with 3.5 kDa) filled with an electrolyte solution for 50 min. Step 2: Next, the electrode was replaced with a platinum rod. The resulting dispersion (from Step 1) was exposed to a current (2.5 A at 10.6 V) for 60 min to conduct extra exfoliation and obtain smaller flakes with a higher degree of oxidation; ultrasonic treatment was used throughout all stages in the work mode (4 s) and after the rest mode (2 s) and maintained the temperature within the range of 25–30 °C. The total volume of the electrolyte used in the synthesis was 800 mL.

Procedure 4: Preparation of aqueous dispersions of graphite oxide by anodic oxidation in separate cathode and anode compartments without pulsed ultrasonic modulations.

Synthesis was carried out in one step with separated cathode and anode compartments (salt bridge filed up with KCl 3.5 M). A current (0.15 A at 30 V) was applied through a graphite anode for 15 h 15 min. The total volumes of electrolytes used in the synthesis were 500 mL (cathode compartment) and 500 mL (anode compartment).

Procedure 5: Preparation of aqueous graphene oxide dispersions by anodic oxidation in separate cathode and anode compartments with pulsed ultrasonic modulations.

The experiment was carried out in separate cathode and anode compartments (salt bridge filed with KCl 3.5M). A current (0.1 A at 30 V) was applied through a graphite anode for 5 h. Ultrasonic treatment was used throughout all stages in the work mode (4 s) and after the rest mode (2 s) and maintained the temperature within the range of 25–30 °C. The total volumes of electrolytes used in the synthesis were 500 mL (cathode compartment) and 500 mL (anode compartment).

Procedure 6: Preparation of aqueous dispersions of graphite oxide by anodic oxidation in a combined cathode–anode compartment without pulsed ultrasonic modulations.

The experiment was carried out in a combined cathode–anode compartment. A current (3 A at 12.3 V) was applied to the graphite electrode for 1.5 h. Ultrasonic treatment was used throughout all stages in the work mode (4 s) and after the rest mode (2 s) and maintained the temperature within the range of 25–30 °C. The total volumes of electrolytes used in the synthesis were (1) 500 mL (cathode compartment) and (2) 500 mL (anode compartment).

#### 3.8.3. Purification Procedures

Procedure 7: Pretreatment of the membrane bag for rinsing.

The required length of the dialysis bag was cut off following the manufacturer’s recommendations. Then, 150 mL 5 mM EDTA solution and 3% H_2_O_2_ were placed in an Erlenmeyer flask (total volume, 250 mL). The thoroughly moistened membrane was immersed in the solution and left with stirring for 5 h. This procedure was carried out to remove metal impurities from the membrane cavities.

Procedure 8: Purification of electrochemically generated graphene oxide aqueous solution.

Purification of the prepared samples was carried out preliminarily by centrifugation (30 min, 4000 rpm) and after dialysis treatment (cellulose membrane, 3.5 kDa [21]). The prepared aqueous dispersion of EGO was placed in a dialysis bag. First, the bag was immersed in 500 mL of a solution of 5 mM EDTA and 3% H_2_O_2_ and left to stir for 5 h. Then, the purification solution was replaced with deionized water and left for mixing, replacing the solvent every 6 h until the electrical conductivity was close to ca. 40–70 μS/cm.

#### 3.8.4. Chemiluminometry Assays

Procedure 9: Chemiluminescent assay for evaluation of superoxide dismutase-like activity in the luminol, Co(II), and H_2_O_2_ system.

The working cobalt(II) solution (20 µM) was prepared by dissolving CoCl_2_×6H_2_O suspension in deionized water. The luminol solution (50 µM) was prepared by dissolving the suspension in a phosphate buffer solution (PBS, pH 7.4); the solution was alkalized for dissolution, then brought to pH 7.4. The hydrogen peroxide solution was prepared by diluting a 60% H_2_O_2_ solution in deionized water. Chemiluminescence was recorded on a 12-channel Lum-1200 instrument at room temperature. Aliquots containing Co(II) and luminol were added to a cuvette containing PBS. A background signal was recorded. Next, an aliquot of hydrogen peroxide was added to the system. After that, EGO was added, and chemiluminescence signals were recorded for 90 min at 25 °C. In all experiments, two replicate measurements were conducted.

Procedure 10: Chemiluminescence assay to estimate pro-oxidant activity in a system with lipid and phospholipid hydroperoxides in the presence of coumarin-334.

Fe(II) ions were used as a model oxidizer. A solution of 20 mM Fe(II) from standard reference material was used. Coumarin 334 solution (5 mM) was prepared by dissolving the sample in a neat preliminary purified DMSO. A solution of linoleic acid (5 mM) was prepared by dissolving an aliquot of the substance in DMSO. A phosphatidylcholine solution (28 mM) was prepared by dissolving the sample in a phosphate buffer solution. Chemiluminescence was recorded on a Lum-100 single-channel chemiluminometer. The background signal of a mixture of PBS, coumarin-334, and analyzed hydroperoxide was recorded for 30–60 s to estimate stationary signals. Next, an aliquot of EGO was injected, and chemiluminescence signals were recorded for 4 min at 25 °C. In all experiments, two replicate measurements were conducted.

## 4. Conclusions

Thus, the electrochemical production of graphene oxide (EGO) is an environmentally friendly method, and it also enables more accurate control of the degree of oxidation of the sample by varying the current and time of exposure to the current. The use of pulsed ultrasonic radiation in the 4/2 s (work/rest) mode makes it possible to increase the dispersion efficiency while reducing the appearance of metal impurities during the production process, and the use of sonication during the electrochemical process significantly controlled the degree of defects in graphene compared to chemically synthesized graphene oxides. This method makes it possible to obtain sedimentation and stable aggregation dispersions (>8 months) exhibiting antioxidant activity, which makes it possible to use EGO in medical and biological industries.

## Figures and Tables

**Table 1:** Generalized experimental conditions for preparations of graphene oxide (EGO) by ultrasound-assisted (US) anodic exfoliation–oxidation.

#	Type of Anode (Stage 1/Stage 2)	Current, A (Stage 1/Stage 2)	Current Density (at the Working Electrode) *j*, mA/cm^2^	Voltage, V (Stage 1/Stage 2)	Espouse Time, min	Procedure Reference Number	Separated Cathode–Anode Compartment	Comment	Brief Result
1	Graphite rod ⌀ = 3.2 mm/Pt wire	2.17/3.37	2.14/7.14	10.2/8.25	15 + 75	Procedure 2	Not used	Anode activation for 10 min using continuous ultrasound treatment	Without the formation of EGO (the anode is stratified)
2	Graphite rod ⌀ = 2 mm/Pt wire	0.53/2.5	0.84/5.30	15.4/10.6	50 + 60	Procedure 3	With membrane	Collected the resulting graphitized foam *	EGO 2
3	Graphite rod ⌀ = 2 mm/Pt wire	0.53/2.5	0.84/5.30	15.4/10.6	50 + 60	Procedure 3	With membrane 10 × 10 cm 3.5 kDa	Collected the solution *	Sample with a negligible ratio of C/O
4	Graphite rod ⌀ = 3.2 mm/n/a	0.15/n/a	0.15/n/a	30/n/a	15 h 15 min	Procedure 4	Salt bridge with 3.5 M KCl	Without ultrasonic treatment	Sample with a negligible ratio of C/O
5	Graphite rod ⌀ = 3.2 mm/n/a	0.1/n/a	0.10/n/a	30/n/a	5 h	Procedure 5	Salt bridge with 3.5 M KCl	n/a *	EGO 5
6	Graphite rod ⌀ = 3.2 mm/n/a	3/n/a	2.96/n/a	12.3/n/a	1.5 h	Procedure 6	Not used	n/a *	EGO 6

*, ultrasonic treatment in modulation mode 4/2 (work/rest) was used everywhere unless otherwise specified; n/a, is not applicable; #, is experiments number.

**Table 2:** Titanium and aluminum content during ultrasonic modulations estimated by ICP–AES.

Type of Amplitude Modulation of Ultrasound	Ultrasound Processing Time (Working Time) (s)	Rest Time (s)	*c*(Ti) *, ppm	*c*(Al) *, ppm	Number of Modulations	Total Time (min)
Continuous mode	30 min	0	36	1.85	0	30
			$\frac{{c(Ti)}_{pulse}}{{c(Ti)}_{continuous}}$	$\frac{{c(Al)}_{pulse}}{{c(Al)}_{continuous}}$		
1/5	2	9.9	280	525	900	180
1/3	2	6	135	245	900	120
1/2	2	4	70	120	900	90
2/3	2	3	55	100	900	75
1	2	2	30	50	900	60
2	4	2	15	20	450	45
3	6	3	12	15	300	45
4	8	2	9.3	13	225	38
5	9	1.8	8.4	11	200	36
10	9	0.9	2.2	3.2	200	33

*_,_ uncertainty is 15%.

**Table 3:** Binding energies and fraction compositions of components (at. %) in C 1s high-resolution XPS spectra on the surface of the samples.

Spectra	Binding Energies, eV	Fraction of Atoms, %			Bond Type
		EGO 5	EGO 2	EGO 6	
C 1s	284.1–284.2	47.0	37.2	0.0	C−C/C−H (sp^2^)
	284.9–285.0	17.6	39.4	74.9	C−C/C−H (sp^3^)
	286.5–286.7	24.8	14.6	14.1	C−O
	287.8–287.9	5.3	4.0	4.6	O−C−O, C=O
	288.8–289.0	5.3	4.8	6.4	O=C−O
	**C, at. %**	74.5	64.0	70.0	
	**O, at. %**	24.6	28.9	22.3	
	**C:O ratio**	4.04	2.95	4.19	

**Table 4:** Zeta potential, EGO flake sizes, and total organic carbon concentration in aqueous dispersions (*n* = 3, *p* = 0.95).

Sample Badge	Procedure	GO Flake Size, nm		PDI	Zeta Potential, mV	*c*_TOC_, ppm	Yield, %
EGO 2	3	270 ± 30	80 ± 10	0.567	−18.7 ± 1.1	80 ± 10	9.2
EGO 5	5	360 ± 35	140 ± 15	0.373	−23.4 ± 1.1	76 ± 10	8.1
EGO 6	5	190 ± 20	140 ± 15	0.580	−22.5 ± 1.1	84 ± 10	9.9

## Data Availability

All inquiries can be directed to the corresponding authors.
